# The tobacco gardens

**Published:** 2012-12-30

**Authors:** Pedro Rovetto

**Affiliations:** Universidad del Valle, Department of Pathology. Cali, Colombia. E-mail: rovettos@hotmail.com

In 2008 it was closed and dismantled the spectacular Duke Gardens near Princeton University. They were created by the famous heiress Doris Duke, in honor of her father, James Buchanan Duke. This last gentleman caused 100-million deaths during the 20th century. The gardens mentioned demonstrated, perhaps trivially, what was stated by philosopher Walter Benjamin: There has never been a document of culture, which is not simultaneously one of barbarism".

"Buck" Duke was the inventor of the modern cigarette. By the end of the 20th century, this astute manufacturer entered the instantly ready-to-smoke tobacco market (without having to roll in small pieces of paper or cut off the cigarette tips) with the automated production of cigarettes. Without a cigarette maker like Carmen from opera by Bizet who rolled a maximum of 200 cigarettes per day, the machine he perfected with a mechanic named Bonsack produced 120,000 "cigarettes" during the same time. Thereby, reaching a oversupply that had to be sold - creating a demand for it.

The solution was cigarette marketing and advertising. These were placed in restaurants, bars, and cigar stores; thus, making them an important part of the worker's period of rest and dining. Although, in principle, they were associated to women of free morals ("Smoking is a great sensual pleasure.While smoking, I await for the man I love ..." sang Sarita Montiel in the 1950s) in a stroke of clever advertising these were transformed into symbols of women's liberation. Toward the late 1920s, young women were seen marching and brandishing their freedom torches, the cigarettes. During the two world wars, cigarettes were distributed to hundreds of thousands of soldiers as part of their daily nutritional ration. During the immediate post-war, packs of Camel and Lucky Strike were the most used Exchange currency in Europe. With all these publicity maneuvers, Mr. Duke and his partners have caused, as we already stated, over 100-million deaths throughout the world; more than Hitler and Stalin together.

How did tobacco come to dominate the human mind in such a way? On the 15th of October 1492, the American aborigines offered Christopher Columbus a bundle of dry tobacco leaves. One month later, two of his seamen reported having seen in Cuba inhabitants from that island inhaling the smoke from those leaves. As part of the Columbian exchange, Europe became aware of the supposed medicinal power and use of tobacco by shamans. After multiple mentions by chroniclers, the French ambassador in Lisbon, Nicot, sent the queen of France, Catherine de Medici, leaves of the plant. Hence, the name nicotine and some suggested that it could have been called "medicine" for Catherine, which would be more appropriate to its original use. By the end of the 21st century, the Chinese had already introduced tobacco to Mongolia and Eastern Siberia - with it managing to go around the world in less than a century. But we could wonder on the popularization of tobacco compared to cocaine, which also occupied a similar niche in the New World's pharmacopoeia.

Goodman, a tobacco historian, offers several answers. Tobacco use was geographically broader (the whole American continent) tan cocaine use (basically, the Inca empire). Besides, tobacco produces moderate excitability followed by a sense of relaxation and cocaine leads to more pronounced euphoria and dysphoria. But there is a reason that brings us back to Duke's merchant strategy; tobacco was introduced in Europe from court to court by physicians and scholars. Its use, then, was distributed to the population mass from the upper classes as a fashion and luxury article. Cocaine, on the contrary, was always associated with impoverished Indians from the highlands who used it to diminish hunger, thirst, and fatigue. Therefore, from the beginning, tobacco was well marketed, as it is currently expressed.

Medical professionals participated in these propaganda efforts; during the 16th century tobacco was introduced as a panacea. During the 20th century, it was sometimes offered as a cure for asthma. With some difficulty, we began recognizing its harmful health effects. English scientist, Hariot, Galileo's predecessor in observing the moon through the telescope, accompanied in 1585 the first English colonists to North America, becoming addicted to tobacco and dying of lips and nasopharyngeal cancer. Today, we still have physicians who smoke, which exemplifies a scandalous historical blindness. Then, to conclude, "Buck" Duke, the honoree in the gardens, is not the only one to blame for those 100-million dead during the last century.

